# Epidemiology of Nontuberculous Mycobacteria in Tuberculosis suspects, Southwest of China, 2017-2022

**DOI:** 10.3389/fcimb.2023.1282902

**Published:** 2023-10-31

**Authors:** Dong-Mei Wang, Hong Liu, Yong-Li Zheng, Yuan-Hong Xu, Yi Liao

**Affiliations:** ^1^ Department of Science and Education Division, Public Health Clinical Center of Chengdu, Chengdu, Sichuan, China; ^2^ Department of Clinical Laboratory Medicine, Chengdu Women’s and Children’s Central Hospital, School of Medicine, University of Electronic Science and Technology of China, Chengdu, China

**Keywords:** epidemiology, TB, drug resistance, NTM, clinical characteristics

## Abstract

**Objectives:**

This study summarizes the epidemiological characteristics, species distribution, and drug sensitivity of clinical nontuberculous mycobacteria (NTM) isolates at the Public Health Clinical Center of Chengdu, China, from January 2017 to December 2022.

**Methods:**

We retrospectively analyzed data from patients with clinically isolated NTM strains. Chi-square analysis assessed the rate of *Mycobacterium* strain isolation over 6 years.

**Results:**

The number of samples tested for *Mycobacterium tuberculosis* (MTB) and/or NTM increased each year, while MTB detection decreased and NTM detection rose significantly each year (P=0.03). The average age of NTM patients was 51 ± 17.53 years, with a 14.1% HIV infection rate. The predominant isolates were *Mycobacterium avium-intracellulare* (MAC) and *M. chelonae*/*M. abscessus*, with 96.4% of cases being of Han ethnicity. Amikacin, moxifloxacin, and clarithromycin were effective against *M. avium* and *M. intracellulare*; linezolid, amikacin, and cefoxitin were effective against *M. chelonae*/*M. abscessus*. Over 90% of NTM cases originated from the respiratory tract.

**Conclusion:**

The NTM isolation rate in Southwest China has risen in recent years, primarily among elderly patients with a high HIV co-infection rate. The main NTM isolates were MAC and *M. chelonae*/*M. abscessus*. Amikacin, moxifloxacin, clarithromycin, and linezolid exhibited strong antibacterial activity against SGM, while amikacin and linezolid displayed relatively better antibacterial activity against RGM. The prevalence of NTM infection may be positively associated with regional economic development and health conditions.

## Introduction

Nontuberculous mycobacteria (NTM) are part of the *Mycobacterium* species, distinct from those in the *M. tuberculosis* complex or *M. leprae*, and typically act as opportunistic pathogens (Falkinham, 1996; Falkinham, 2002). This term encompasses around 200 distinct *Mycobacterium* species, with new species continually emerging (Zhou et al., 2020). In recent years, both in China and globally, NTM infections have been on the rise (Wang et al., 2019; Liu et al., 2021; Thornton et al., 2021). Among clinical laboratories in China, the *Mycobacterium* avium-intracellulare complex (MAC) and the *M. chelonae*/*M. abscessus* complex are the two most commonly encountered NTM complexes, and they are among the most drug-resistant species (Wang et al., 2016; Wang et al., 2019; Guo et al., 2021). However, limited studies have explored NTM drug susceptibility due to small sample sizes or restricted antibiotic types being tested. Therefore, this study aims to summarize NTM identification and drug sensitivity data from the largest sample size in a major central city in Southwest China. This information can provide valuable insights for the prevention and treatment of NTM diseases.

## Methods

### Study population and diagnostic criteria

This study included all patients with culture-positive NTM infections treated at the Public Health Clinical Center of Chengdu (PHCC) in Sichuan Province, China, from January 2017 to December 2022. During the 6-year study period, a total of 126,368 suspected mycobacterial infections were cultured using the BACTEC™ MGIT 960 System (Becton Dickinson & Co., NJ, USA), resulting in 26,510 *Mycobacterium tuberculosis* (MTB) culture-positive cases and 587 NTM culture-positive cases. Diagnosis and categorization of NTM-infected patients were based on the 2012 and 2020 NTM Diagnosis and Treatment Expert Consensus (Editorial Board of CSTB, 2016; Tuberculosis Branch of Chinese Medical Association, 2020), the Clinical Diagnosis and Treatment Guidelines for Tuberculosis in China (Chinese Medical Association, 2005), and the updated guidelines from the World Health Organization. Diagnosis of human immunodeficiency virus (HIV) followed the Chinese HIV and HIV Infection Diagnostic Criteria (WS293–2008) (From the Centers for Disease Control and prevention, 1993).

### Bacterial strains culture, identification, and drug sensitivity

We employed the BACTEC™ MGIT 960 System for culturing mycobacteria. Extrapulmonary samples (such as pleural fluid, spinal fluid, and lymph nodes) were obtained through lumbar puncture, pleural tap, fine needle aspiration, lymph node biopsy, and other procedures (Wang et al., 2017). Initial identification of NTM bacteria primarily relied on the MPT 64 antigen detection (Colloidal Gold immunochromatography) or polymerase chain reaction (PCR) methods. Subsequently, P-nitrobenzoic acid (PNB) and thiophene-2-carboxylic acid hydrazide (TCH) were employed for NTM revalidation using the MicroDST ™ (Yinke AUTOBIO Diagnostics Co., Ltd, Zhuhai, China) approach (Cao et al., 2021). Further identification of NTM species/complexes was conducted using Genechip, following the manufacturer’s instructions (CapitalBio Corp., Chengdu, China). Strains that couldn’t be identified via genechip were subjected to analysis using matrix-assisted laser desorption/ionization time-of-flight mass spectrometry (Microflex LT; Bruker Daltonics, Bremen, Germany) or 16S rDNA sequencing.

The Clinical and Laboratory Standards Institute (CLSI) recommends the microplate dilution method for *in vitro* drug sensitivity testing of some Slow-Growing Mycobacteria (SGMs) and Rapid-Growing Mycobacteria (RGMs). However, the exact Minimum Inhibitory Concentration (MIC) values for the strains require comprehensive evaluation and adjustment for clinical practice (Clinical and Laboratory Standards Institute (CLSI), [[NoYear]]). In this study, drug resistance tests (DST) for culture-positive NTM isolates were conducted using MicroDST ™ (Yinke AUTOBIO Diagnostics Co., Ltd, Zhuhai, China). The MIC was defined as the lowest drug concentration inhibiting visible growth of the tested isolates. MIC breakpoints and sensitivity/resistance determinations were interpreted following reagent instructions, and the protocol was executed in accordance with the manufacturer’s recommendations (Wang et al., 2020). We employed a total of 13 antimicrobial agents in this study, including rifampicin (1, ***4***, 6, 16 μg/mL), clarithromycin (0.5, 4, ***16***, 64 μg/mL), imipenem/cilastatin (IPM/CS) (0.5, 4, 16, ***64*** μg/mL), linezolid (0.5, 2, 8, ***32*** μg/mL), amikacin (1, 4, 16, ***64*** μg/mL), ethambutol (2.5, ***5***, 10, 20 μg/mL), and rifabutin (0.5, ***2***, 8, 32–10 μg/mL), at four concentrations. Cefoxitin (4, 16, 32, 40, 64, ***80***, 128, 160 μg/mL), tobramycin (0.5, 1, 2, 4, 8, ***16***, 32, 64 μg/mL), moxifloxacin (0.125, 0.25, 0.5, 1, 2, 4, ***8***, 16 μg/mL), doxycycline (0.5, 1, 4, ***8***, 16, 32, 64, 128 μg/mL), minocycline (0.5, 1, 4, ***8***, 16, 32, 64, 128 μg/mL), and sulfamethoxazole (8, 16, 32, 64, ***80***, 128, 160, 256 μg/mL) were employed at eight concentrations. Bold and italicized values in the aforementioned drug concentrations represent the resistance breakpoint for each drug. For NTM from the same case, site, and type, the DST results from the initial culture were considered. Monitoring was conducted using control strains H37Rv (ATCC 25618) and *M. smegmatis* (CGMCC 1.2621).

### Laboratory quality control

External quality assessment (EQA) was carried out for smear, culture, and DST at the Innovation Alliance for TB Diagnosis and Treatment in Beijing, China. Additionally, a blinded retesting of approximately 10% of isolates from the study laboratory was conducted by a specialized Centers for Disease Control and Prevention.

### Statistical analysis

Data were analyzed using SPSS Statistics Client 19.0 (SPSS Inc., IL, USA). Normally distributed measurement data were presented as means, while categorical variables were expressed as numbers and percentages. Chi-square (χ^2^) analysis was employed to assess variations in the *Mycobacterium* strain rate, age, and sex ratio over five years. Statistical significance was set at P < 0.05.

### Ethics approval and consent to participate

This study received approval from the Ethics Committee of PHCC (Approval No. 2017Y025). All patient information used in this study was routinely collected through the mandatory notification system. The requirement for informed consent was waived by the ethics committee.

## Results

### Demographic and clinical characteristics

**Figure 1:**
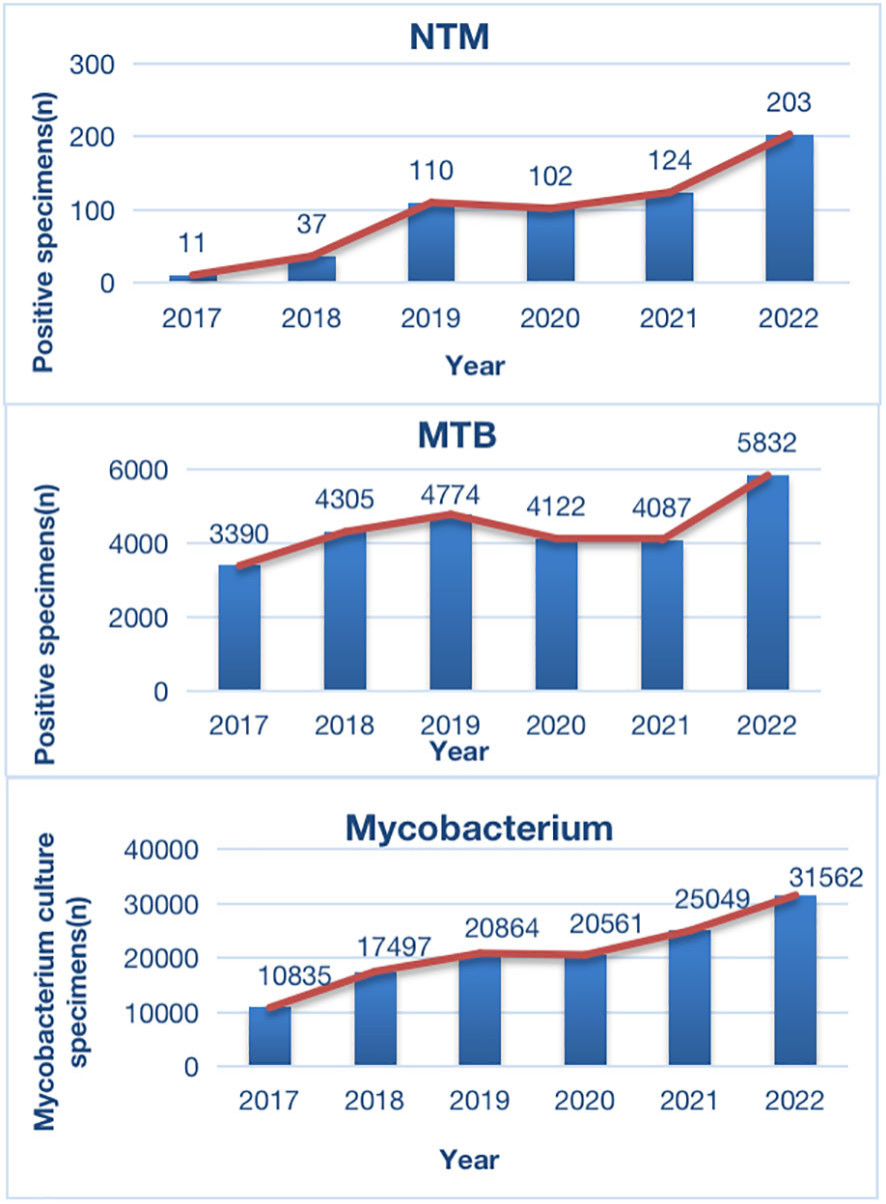
Distribution of Positive Specimens in *Mycobacterium* Culture During 2017-2021. MTB, *Mycobacterium* tuberculosis; NTM, non-tuberculous mycobacteria.

**Figure 2:**
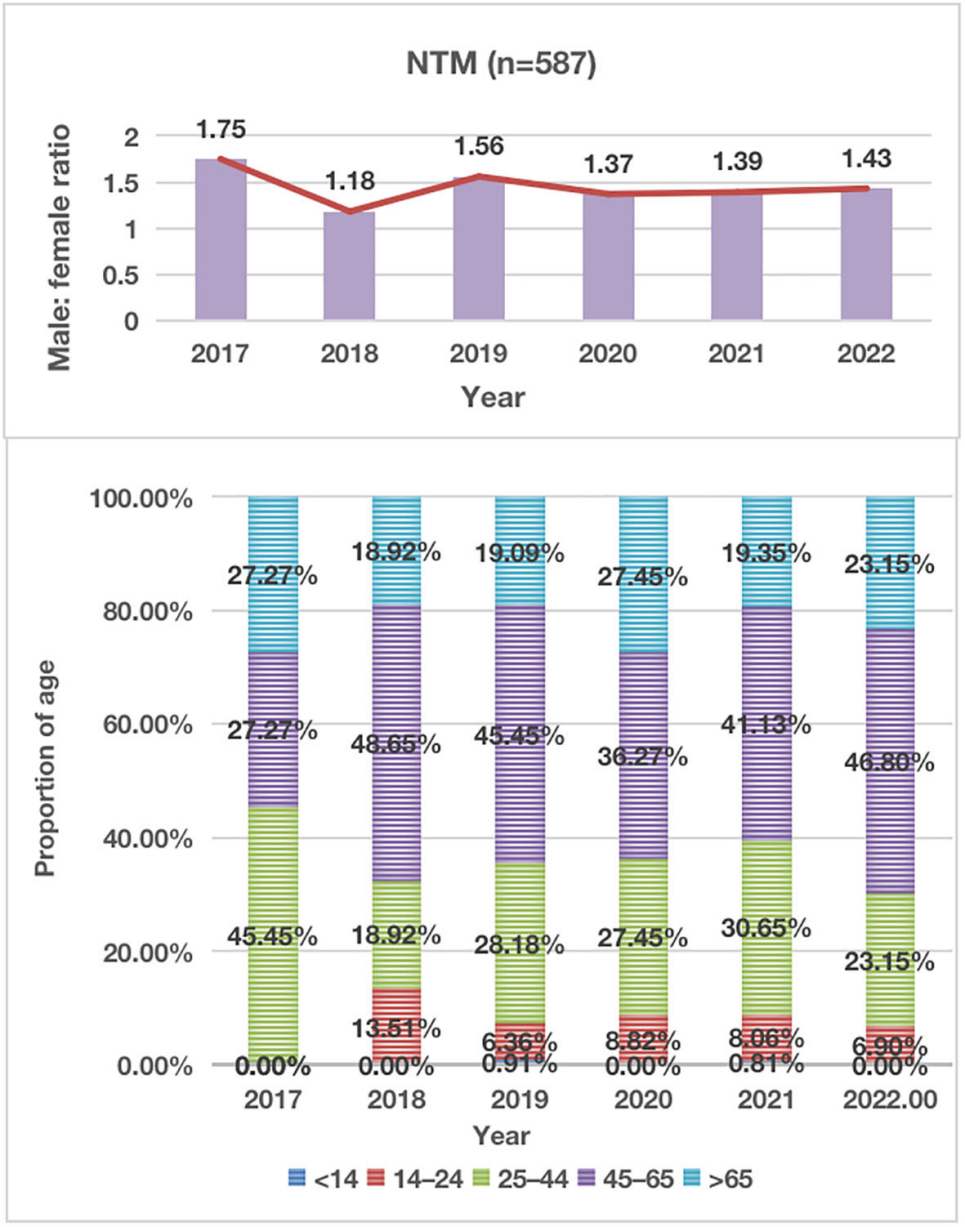
Distribution of Male-to-Female Ratio and Proportion of Age in NTM Patients During 2017-2021. NTM, nontuberculous mycobacteria.

From January 2017 to December 2022, a total of 126,368 non-repeated clinical specimens with suspected mycobacterial infections were cultured at PHCC. Among these specimens, 26,510 (21.0%) tested positive in culture. MTB was detected in 25,923 (97.8%) positive samples, while NTM was found in 587 (2.2%) samples. Among all NTM cases, 13 were co-infected with both MTB and NTM (**Figure 1**; **Table 1**). Over the 6-year study period, a significant increase was observed in the number of samples tested for MTB and/or NTM each year, corresponding to a significant annual rise in NTM detection (χ^2 = ^18.01, P=0.03; **Figure 1**). The mean age of the 587 NTM patients was 51 ± 17.53 years (range: 13–88 years). Within this subgroup, 349/587 (59.5%) were males, and 238/587 (40.5%) were females, with a male-to-female ratio of 1.5. There was no significant difference in the male-to-female ratio over the 6-year period (χ^2 = ^0.71, P>0.05; **Figure 2**). The majority of NTM-infected cases were middle-aged and elderly patients, and the proportion of each age group showed no significant difference over the 6 years (χ2 = 11.16, P>0.05; as shown in **Figure 2**; **Table 1**).

**Table 1:** General characteristics among patients with NTM disease (n=587).

Category		No. of subjects (NTM, n=574)	No. of subjects (NTM/MTB*, n=13)	No. of subjects (% ) Total (n=587)
Sex				
	Male	341	8	349 (59.5)
	Female	233	5	238 (40.5)
Age				
	Mean ± SD; years (range)	52 ± 18.47 (13-88)	43 ± 21.26 (26-80)	51 ±17.53
	<14	2	0	2 (0.3)
	14–24	45	0	45 (7.7)
	25–44	149	7	156 (26.6)
	45–65	250	4	254 (43.3)
	>65	128	2	130 (22.2)
**Chinese Ethnic minorities**	Han	554	12	566 (96.4)
	Tibetan	17	1	18 (3.1)
	Others	3	0	3 (0.5)
Co-infectious disease				
	AIDS	82	1	83 (14.1)
	Syphilis	3	0	3 (0.5)
	Combined with Diabetes	28	1	29 (4.9)
	Combined with hypertension	9	0	9 (1.5)
	Hepatitis E	1	0	1 (0.2)
	Hepatitis B	12	0	12 (2.0)
Adverse drug reaction				
	Liver dysfunction	80	2	82 (14.0)
	Hyperuricemia	24	0	24 (4.1)
	Leucopenia	19	0	19 (3.2)
	Thrombocytopenia	8	0	8 (1.4)
	Drug eruption	7	0	7 (1.2)

* NTM/MTB, among 587 cases of NTM infection, 13 cases of MTB and NTM co-infection.

Twenty-one (3.6%) patients belonged to ethnic minorities. Among the 587 patients, 93 (15.8%) had at least one co-infectious disease: HIV infection in 83 (14.1%), diabetes mellitus in 29 (4.9%), hepatitis B virus in 12 (2.0%), and hypertension in 9 (1.5%), syphilis in 3 (0.5%), and hepatitis E virus in 1 (0.2%). Additionally, 99 (16.8%) patients experienced at least one adverse drug reaction: liver dysfunction in 82 (14.0%), hyperuricemia in 24 (4.1%), leucopenia in 19 (3.2%), thrombocytopenia in 8 (1.4%), and drug eruption in 7 (1.2%) patients.

### NTM species identification

**Figure 3:**
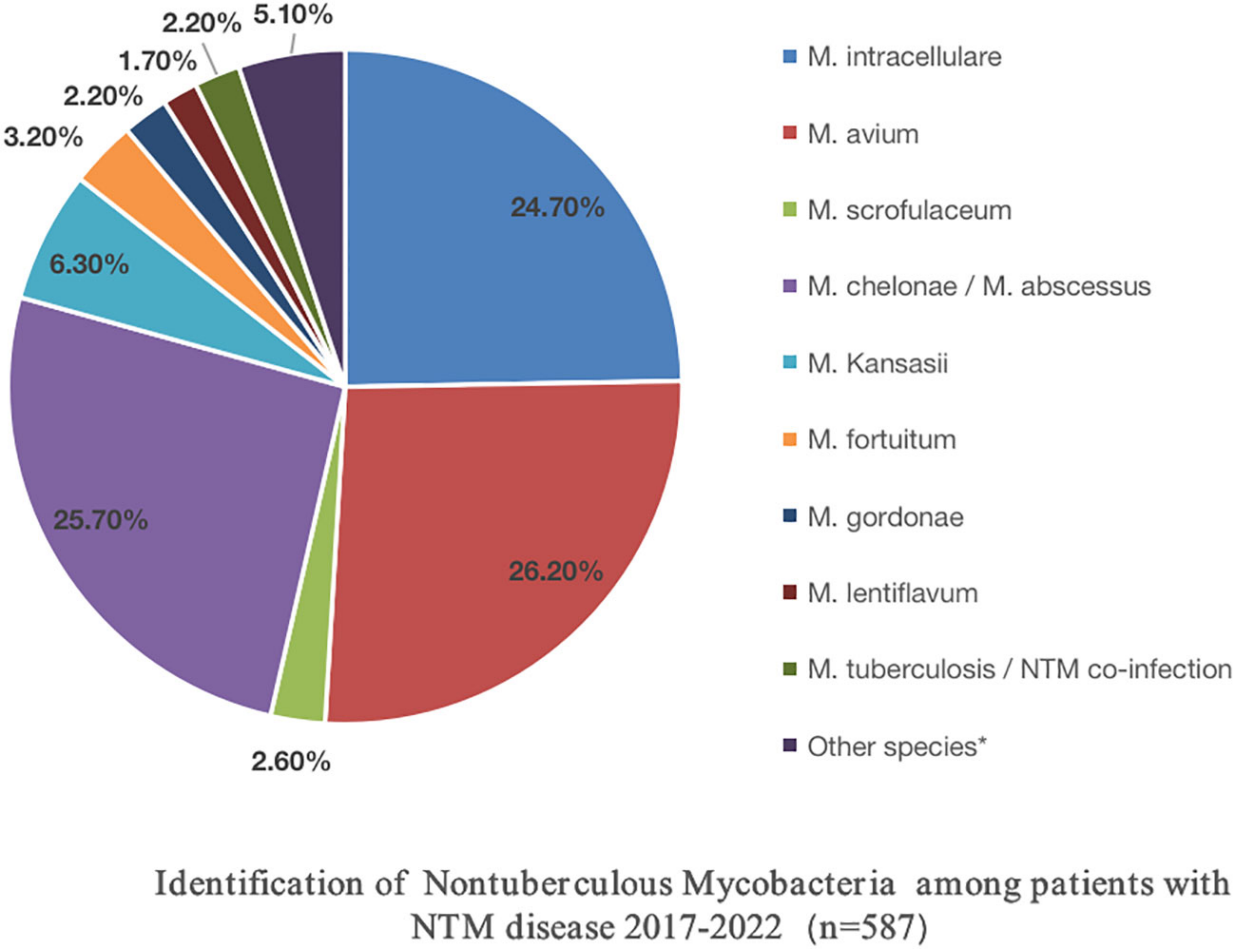
Identification of Nontuberculous Mycobacteria Among Patients with NTM Disease (n=587).

The DNA microarray chip identified NTM species as follows: *M. avium* in 154 (26.2%), *M. chelonae*/*M. abscessus* in 151 (25.7%), *M. intracellulare* in 145 (24.7%), *M. kansasii* in 37 (6.3%), *M. fortuitum* in 19 (3.2%), *M. scrofulaceum* in 15 (2.6%), *M. gordonae* in 13 (2.2%), *M. lentiflavum* in 10 (1.7%), and mixed infections of MTB and NTM in 13 (2.2%). Additionally, 30 specimens initially identified as other *Mycobacterium* spp. by the Genechip (CapitalBio Corporation) included *M. szulgai, M. malmoense, M. terrae, M. peregrinum, M. margueri, M. phlei, M. septicum, M. marseillense, M. shigaense, M. xenopi, M. simiae*, and *M. lentil*, identified through 16S rDNA sequencing or matrix-assisted laser desorption/ionization time-of-flight mass spectrometry systems. Moreover, a significant rise in NTM detection was observed from 2017 to 2022 (**Table 2**; **Figure 3**).

**Table 2:** Identification of Nontuberculous Mycobacteria among patients with NTM disease (n=587).

Species	2017	2018	2019	2020	2021	2022	Total (%)
***M. intracellulare***	4	13	33	17	27	51	145 (24.7)
***M. avium***	0	9	29	31	32	53	154 (26.2)
***M. scrofulaceum***	0	0	1	2	2	10	15 (2.6)
***M. chelonae / M. abscessus***	5	11	29	23	31	52	151 (25.7)
***M. Kansasii***	1	1	8	5	10	12	37 (6.3)
***M. fortuitum***	0	0	3	4	5	7	19 (3.2)
***M. gordonae***	0	0	2	5	4	2	13 (2.2)
***M. lentiflavum***	0	0	0	3	3	4	10 (1.7)
***M. tuberculosis / NTM co-infection***	0	0	1	7	2	3	13 (2.2)
Other species*	1	3	4	7	6	9	30 (5.1)
**Total (%)**	11	37	110	104	122	203	587

Other species* include *M. szulgai, M. malmoense, M. terrae, M.peregrinum, M. margueri, M. phlei, M. septicum,M. marseillense,M. shigaense, M. xenopi, M. simiae* and *M. lentil*.

### Drug resistance of the NTM species

Out of the 587 patients with NTM disease, antimicrobial susceptibility testing was conducted for 113, as depicted in **Table 3**. Among the Slowly Growing Mycobacteria (SGM), amikacin (high-level resistance ≥ 64 μg/mL) demonstrated the highest activity against *M. avium*, with a resistance rate of 2/29 (6.9%). Moxifloxacin (high-level resistance ≥ 8 μg/mL) exhibited the highest activity against *M. intracellulare*, with a resistance rate of 2/27 (7.4%). Clarithromycin (high-level resistance ≥ 16 μg/mL) displayed effective antibacterial effects on both *M. avium* and *M. intracellulare*, with drug resistance rates of 5/29 (17.2%) and 6/27 (22.2%), respectively. In this study, all six strains of *M. kansasii* were completely sensitive to clarithromycin, linezolid (high-level resistance ≥ 32 μg/mL), amikacin, and moxifloxacin. Eight strains of *M. gordonae* showed complete sensitivity to rifampicin (high-level resistance ≥ 4 μg/mL), clarithromycin, linezolid, amikacin, cefoxitin (high-level resistance ≥ 80 μg/mL), and moxifloxacin. Linezolid, amikacin, tobramycin (high-level resistance ≥ 16 μg/mL), and moxifloxacin also exhibited strong antibacterial activity against *M. scrofulaceum*.

**Table 3:** Number of Nontuberculous Mycobacteria clinical strains resistant to drugs in vitro experiments.

Antimicrobial agents	No of NTM species/complex (%)									
	SGM (n=73)						RGM (n=40)			
	*M. avium*(n=29)	*M*. *intracellulare*(n=27)	*M*. *kansasii*(n=6)	*M. gordonae*(n=8)	*M. scrofulaceum*(n=3)	Total (%)	*M. chelonae/ M. abscessus*(n=35)	*M. lentiflavum*(n=2)	*M. fortuitum*(n=3)	Total (%)
**Rifampicin**	14 (48.3)	8 (29.6)	3 (50.0)	0 (0)	2 (66.7)	27 (37.0)	33 (94.3)	0 (0.0)	3 (100.0)	36 (90.0)
**Clarithromycin**	5 (17.2)	6 (22.2)	0 (0)	0 (0)	2 (66.7)	13 (17.8)	22 (62.9)	0 (0.0)	3 (100.0)	25 (62.5)
**Imipenem/ cilastatin ( IPM/ CS)**	29 (100.0)	26 (96.3)	6 (100.0)	8 (100.0)	3 (100.0)	72 (98.6)	35 (100.0)	2 (100.0)	3 (100.0)	40 (100.0)
**linezolid**	11 (37.9)	11 (40.7)	0 (0)	0 (0)	1 (33.3)	23 (31.5)	15 (42.9)	0 (0.0)	0 (0.0)	15 (37.5)
**Amikacin**	2 (6.9)	5 (18.5)	0 (0)	0 (0)	1 (33.3)	8 (11.0)	18 (51.4)	0 (0.0)	0 (0.0)	18 (45.0)
**Ethambutol**	19 (65.5)	10 (37.0)	1 (16.7)	2 (25.0)	2 (66.7)	34 (46.6)	33 (94.3)	1 (50.0)	2 (66.7)	36 (90.0)
**Rifabutin**	19 (65.5)	15 (55.6)	2 (33.3)	1 (12.5)	2 (66.7)	39 (53.4)	33 (94.3)	1 (50.0)	3 (100.0)	37 (92.5)
**Cefoxitin**	19 (65.5)	17 (63.0)	6 (100.0)	0 (0)	2 (66.7)	44 (60.3)	20 (57.1)	1 (50.0)	0 (0.0)	21 (52.5)
**Tobramycin**	7 (24.1)	8 (29.6)	4 (66.7)	7 (87.5)	1 (33.3)	27 (37.0)	33 (94.3)	2 (100.0)	1 (33.3)	36 (90.0)
**Moxifloxacin**	9 (31.0)	2 (7.4)	0 (0)	0 (0)	1 (33.3)	12 (16.4)	29 (82.9)	0 (0.0)	0 (0.0)	29 (72.5)
**Doxycycline**	29 (100.0)	25 (92.6)	6 (100.0)	6 (75.0)	3 (100.0)	69 (94.5)	35 (100.0)	1 (50.0)	2 (66.7)	38 (95.0)
**Minocycline**	28 (96.6)	25 (92.6)	2 (33.3)	5 (62.5)	3 (100.0)	63 (86.3)	35 (100.0)	2 (100.0)	2 (66.7)	39 (97.5)
**Sulfamethoxazole**	27 (93.1)	25 (92.6)	4 (66.7)	7 (87.5)	3 (100.0)	66 (90.4)	34 (97.1)	2 (100.0)	3 (100.0)	39 (97.5)

RGM, rapidly growing non-tuberculous mycobacteria; SGM, slowly growing non-tuberculous mycobacteria. rifampicin (1, 4, 6, 16 μg/mL), clarithromycin (0.5, 4, 16, 64 μg/mL), imipenem/cilastatin (IPM/CS) (0.5, 4, 16, 64 μg/mL), linezolid (0.5, 2, 8, 32 μg/mL), amikacin (1, 4, 16, 64 μg/mL), ethambutol (2.5, 5, 10, 20 μg/mL), and rifabutin (0.5, 2, 8, 32–10 μg/mL), at four concentrations, while cefoxitin (4, 16, 32, 40, 64, 80, 128, 160 μg/mL), tobramycin (0.5, 1, 2, 4, 8, 16, 32, 64 μg/mL), moxifloxacin (0.125, 0.25, 0.5, 1, 2, 4, 8, 16 μg/mL), doxycycline (0.5, 1, 4, 8, 16, 32, 64, 128 μg/mL), minocycline (0.5, 1, 4, 8, 16, 32, 64, 128 μg/mL), and sulfamethoxazole (8, 16, 32, 64, 80, 128, 160, 256 μg/mL) was used at eight concentrations.The bold and italic markings in the above drug concentrations indicate the resistance breakpoint of each drug.

Regarding the Rapidly Growing Mycobacteria (RGM), linezolid, amikacin, and cefoxitin were the most effective agents against *M. chelonae*/*M. abscessus*. Rifampicin, clarithromycin, linezolid, amikacin, and moxifloxacin showed complete sensitivity in two strains of *M. lentiflavum*, while linezolid, amikacin, cefoxitin, and moxifloxacin demonstrated complete sensitivity in three strains of *M. fortuitum*.

### NTM species distribution from different specimen types

Among the 113 NTM strains tested for drug sensitivity in this study, over 90% were isolated from respiratory tract samples [sputum 90/113 (79.6%), bronchoalveolar lavage fluid 12/113 (10.6%)]. Cerebrospinal fluid contributed 7/113 (6.2%) samples, while lymph nodes, the digestive tract, and pleural effusion each provided 2/113 (1.8%), 1/113 (0.9%), and 1/113 (0.9%) samples, respectively. The primary NTM strains isolated from these six tissue sources were *M. chelonae*/*M. abscessus* and MAC.

## Discussion

According to recent reports from China and other countries, there has been a gradual increase in the incidence of NTM infection and laboratory isolation rates (Wang et al., 2016; Wang et al., 2019; Guo et al., 2021; Liu et al., 2021; Thornton et al., 2021). NTMs are inherently resistant to many anti-TB drugs (ATDs), and treatment plans depend on factors such as the NTM species, infection site, and the severity of the infection (Lee et al., 2015; Daley et al., 2020; Gopalaswamy et al., 2020). The prevalence of NTM strains varies among different regions and populations. For instance, the most common NTM isolates reported in various countries include *M. kansasii* and MAC in Poland (Przybylski et al., 2023), MAC and *M. gordonae* in Italy (Giannoni et al., 2023), *M. fortuitum*, and M. simiae in Iran (Tarashi et al., 2023), and MAC, *M. abscessus*, and *M. kansasii* in Turkey (Babalik et al., 2023). In Switzerland, common NTM isolates include *M. avium* and *M. gordonae* (Vongthilath-Moeung et al., 2022). However, information regarding NTM isolates and drug resistance profiles in southwest China has been scarce. Therefore, accurately understanding the NTM epidemic and drug resistance situation is crucial for early differential diagnosis and treatment of TB and NTM diseases.

In southwest China, there has been limited data on NTM infections. Our preliminary study discussed only a small sample of NTM-infected individuals in Southwest China, while a few studies have reported on large samples of clinically NTM-infected individuals and the dynamic sensitivity of NTM to multiple antibiotics in China and worldwide (Jeong et al., 2017; Zhou et al., 2020; Maya et al., 2022). In this study, we conducted a systematic analysis of NTM clinical infection cases in Southwest China over the past six years. The results indicated a rising trend in the number of patients visiting PHCC for mycobacteria culture evaluation each year from 2017 to 2022. This trend may be attributed to increased public awareness of healthcare in recent years and the expanding reach of PHCC in Southwest China. However, in 2020, there was a slight decline in this trend, likely due to the initial COVID-19 pandemic control measures, which resulted in reduced public mobility and hospital visits. Nevertheless, the trend resumed its upward trajectory after 2021. Moreover, among mycobacteria-positive cultures, the count of MTB cases increased from 2017 to 2019 and then gradually decreased until 2020. This trend is consistent with the recent control measures for TB, which have led to decreasing incidence and mortality rates each year (World Health Organization, 2021). Notably, the number of isolated clinical NTM cases showed a continuous upward trend from 2017 to 2022, rising from 11 cases in 2017 to 203 cases in 2022. This finding aligns with reports of increasing NTM infection cases worldwide and the observation that laboratory isolation rates have been on the rise each year (López-Roa et al., 2020).

In this study, NTM infections were primarily observed in middle-aged and elderly individuals, with those aged over 45 accounting for 65.5% of the cases. This is in contrast to our previous studies, where the majority of MTB infections were in middle-aged and young individuals (Wang et al., 2017; López-Roa et al., 2020; Wang et al., 2020). The co-infection rate of acquired immune deficiency syndrome (AIDS) with NTM cases was 14.1%, slightly higher than the 11.5% reported in our previous study (Wang et al., 2019). This increase may be linked to the rising incidence of AIDS cases in southwest China in recent years (Zhang et al., 2015; Wang et al., 2016; Yang et al., 2018). Additionally, some NTM cases were complicated by varying degrees of diabetes and hypertension, indicating that middle-aged and elderly patients with underlying pulmonary diseases, HIV, and compromised immunity are risk factors for NTM infections.

In our previous study, we observed that MTB infections accounted for a significant proportion of ethnic minorities, particularly among children with TBM in Southwest China (Wang et al., 2020). However, in this study, 96.4% of NTM-infected patients were of Han ethnicity from central cities, with minority groups representing a small subset of the infected individuals. This pattern contrasts with the MTB-infected population model in southwest China, suggesting a positive correlation between NTM infection and the regional level of economic development and sanitation conditions.

Among the 587 NTM cases in this study, the top three isolated strains were *M. avium*, *M. chelonae*/*M. abscessus*, and *M. intracellulare*, followed by *M. kansasii*. These clinical strains mirrored the domestic epidemic trend (Jing et al., 2012; Wu et al., 2014; Tan et al., 2018; Liu et al., 2019). Among the 113 cases that underwent *in vitro* drug sensitivity testing, 90.3% of the strains originated from the respiratory tract, followed by cerebrospinal fluid, lymph nodes, the gastrointestinal tract, and pleural effusion. Intriguingly, MAC and *M. chelonae*/*M. abscessus* were the predominant strains in various tissues, followed by *M. kansasii*, *M. fortuitum*, *M. scrofulaceum*, *M. gordoniae*, and *M. lentiflavum*.

NTM naturally exhibits resistance to various anti-TB drugs, and its clinical isolation rate is relatively low. Furthermore, the emergence of NTM resistance poses a significant challenge in clinical treatment. Currently, research on NTM drug resistance is limited, with only a few reports on the sensitivity of NTM to various antibiotics in China, often based on small sample sizes or focused on a single antibiotic (Lan et al., 2011; Li et al., 2017). In this study, we conducted comprehensive *in vitro* sensitivity tests on 113 NTM strains with 13 antibiotics. The results revealed that clarithromycin exhibited significant antibacterial efficacy against *M. avium* and *M. intracellulare*, with drug resistance rates of 17.2% and 22.2%, respectively. The average drug resistance rate among SGM was 17.8%, while for RGM, it was 62.5%. Aminoglycosides are commonly used and effective drugs for NTM treatment. In our study, amikacin demonstrated potent antibacterial effects on *M. avium* and *M. intracellulare*, with low drug resistance rates of 6.9% and 18.5%, respectively. The antibacterial activity of amikacin against MAC was superior to that of clarithromycin. Notably, amikacin also displayed good antibacterial efficacy against both SGM and RGM, with drug resistance rates of 13.7% and 45.0%, respectively. In addition to its effectiveness against MAC, tobramycin’s antibacterial activity against *M. chelonae*/*M. abscessus* was notable at 94.3%, although it differed from amikacin at 51.4%. This discrepancy may be attributed to variations in ethnicity, geographical regions, or clinical isolates. While aminoglycosides demonstrate potent antibacterial activity against NTM, their prolonged use can lead to hepatorenal toxicity and ototoxicity. Therefore, these drugs should be used judiciously in clinical practice. Additionally, in this study, the fluoroquinolone moxifloxacin exhibited robust antibacterial activity against SGM but showed weaker effectiveness against RGM.

## Conclusions

The isolation rate of NTM in southwest China has shown an increasing trend in recent years. The majority of infected cases involve elderly patients, and there has been an elevated proportion of individuals with HIV infection. The predominant clinical isolates are MAC and *M. chelonae*/*M. abscessus*, followed by *M. kansasii* and *M. fortuitum*. Among the tested antibiotics, amikacin, moxifloxacin, clarithromycin, and linezolid demonstrated effective antibacterial activity against SGM, whereas linezolid and amikacin exhibited relatively better antibacterial activity against RGM. The incidence of NTM infection may be positively correlated with the level of regional economic development and healthcare conditions.

## Limitations

One significant limitation of this study is the extended treatment cycle required for NTM infections (Lee et al., 2015; Daley et al., 2020; Gopalaswamy et al., 2020), often exceeding one year or even longer. Consequently, many cases experience issues such as loss to follow-up, poor treatment outcomes, and a high recurrence rate. Although follow-up observations are ongoing for some cases to assess treatment efficacy, the data collection for these cases is incomplete. Future research should aim to provide more comprehensive and valuable reference information for clinical use.

## Data availability statement

The original contributions presented in the study are included in the article/supplementary material, further inquiries can be directed to the corresponding author/s.

## Ethics statement

The studies involving humans were approved by This study was approved by the Ethics Committee of PHCC [2017Y] 025. The studies were conducted in accordance with the local legislation and institutional requirements. Written informed consent for participation was not required from the participants or the participants’ legal guardians/next of kin in accordance with the national legislation and institutional requirements.

## Author contributions

DW: Conceptualization, Data curation, Funding acquisition, Resources, Supervision, Writing – original draft, Writing – review & editing. HL: Data curation, Investigation, Resources, Writing – review & editing. YZ: Data curation, Investigation, Supervision, Writing – review & editing. YX: Data curation, Investigation, Writing – review & editing. YL: Data curation, Project administration, Supervision, Writing – original draft, Writing – review & editing.
